# Giant Seismites and Megablock Uplift in the East African Rift: Evidence for Late Pleistocene Large Magnitude Earthquakes

**DOI:** 10.1371/journal.pone.0129051

**Published:** 2015-06-04

**Authors:** Hannah Louise Hilbert-Wolf, Eric M. Roberts

**Affiliations:** Department of Earth and Oceans, James Cook University, Townsville, Queensland, Australia; University of Stellenbosch, SOUTH AFRICA

## Abstract

In lieu of comprehensive instrumental seismic monitoring, short historical records, and limited fault trench investigations for many seismically active areas, the sedimentary record provides important archives of seismicity in the form of preserved horizons of soft-sediment deformation features, termed seismites. Here we report on extensive seismites in the Late Quaternary-Recent (≤ ~ 28,000 years BP) alluvial and lacustrine strata of the Rukwa Rift Basin, a segment of the Western Branch of the East African Rift System. We document examples of the most highly deformed sediments in shallow, subsurface strata close to the regional capital of Mbeya, Tanzania. This includes a remarkable, clastic ‘megablock complex’ that preserves remobilized sediment below vertically displaced blocks of intact strata (megablocks), some in excess of 20 m-wide. Documentation of these seismites expands the database of seismogenic sedimentary structures, and attests to large magnitude, Late Pleistocene-Recent earthquakes along the Western Branch of the East African Rift System. Understanding how seismicity deforms near-surface sediments is critical for predicting and preparing for modern seismic hazards, especially along the East African Rift and other tectonically active, developing regions.

## Introduction

Earthquakes not only trigger geohazards such as surface ruptures, tsunamis, and landslides, but are also linked to significant, catastrophic soft-sediment deformation. Despite recent events associated with devastating liquefaction and fluidization of near-surface sediments, such as the 2011 New Zealand and Japan earthquakes and the ongoing Lusi mud eruptions in Indonesia, the dangers to life and infrastructure from soft-sediment deformation are often overlooked. In 1910 7.5 million people lived in Tanzania when the most powerful earthquake in Africa of the twentieth century (M_s_ 7.4) struck the Lake Rukwa region, collapsing houses, initiating standing waves in nearby water bodies, causing ground deformation, and triggering liquefaction and fluidization of saturated subaerial and submarine deposits [1, 2]. By 2050 roughly 138 million people will live in Tanzania [3], largely in constructed urban environments. This growth particularly affects the seismically active rift valleys of East Africa, where people concentrate near productive rift lakes and volcanic soils, on substrate that is susceptible to liquefaction and seismite generation and preservation [4].

A combination of approaches to investigate prehistoric seismicity, such as archaeoseismic research, seismic-stratigraphic correlation of event horizons, and characterization of soft-sediment deformation features, are vital for constraining earthquake recurrence intervals and magnitude [4, 5]. In Africa, historical records alone can limit the recognition of long-term earthquake trends due to deficiencies in station numbers, global station distribution, epicentral accuracy, and short instrumental coverage period of only the last ~100 years. Given the potential societal impacts, understanding sediment responses to earthquake activity is an underappreciated aspect of seismic hazard evaluation. Additionally, the ability to recognize and document large-scale soft-sediment deformation and injectite features in outcrop is increasingly advantageous in light of heightened interest in the association of large-scale sandstone intrusions and hydrocarbons.

## Seismicity in the Rukwa Rift

**Fig 1:**
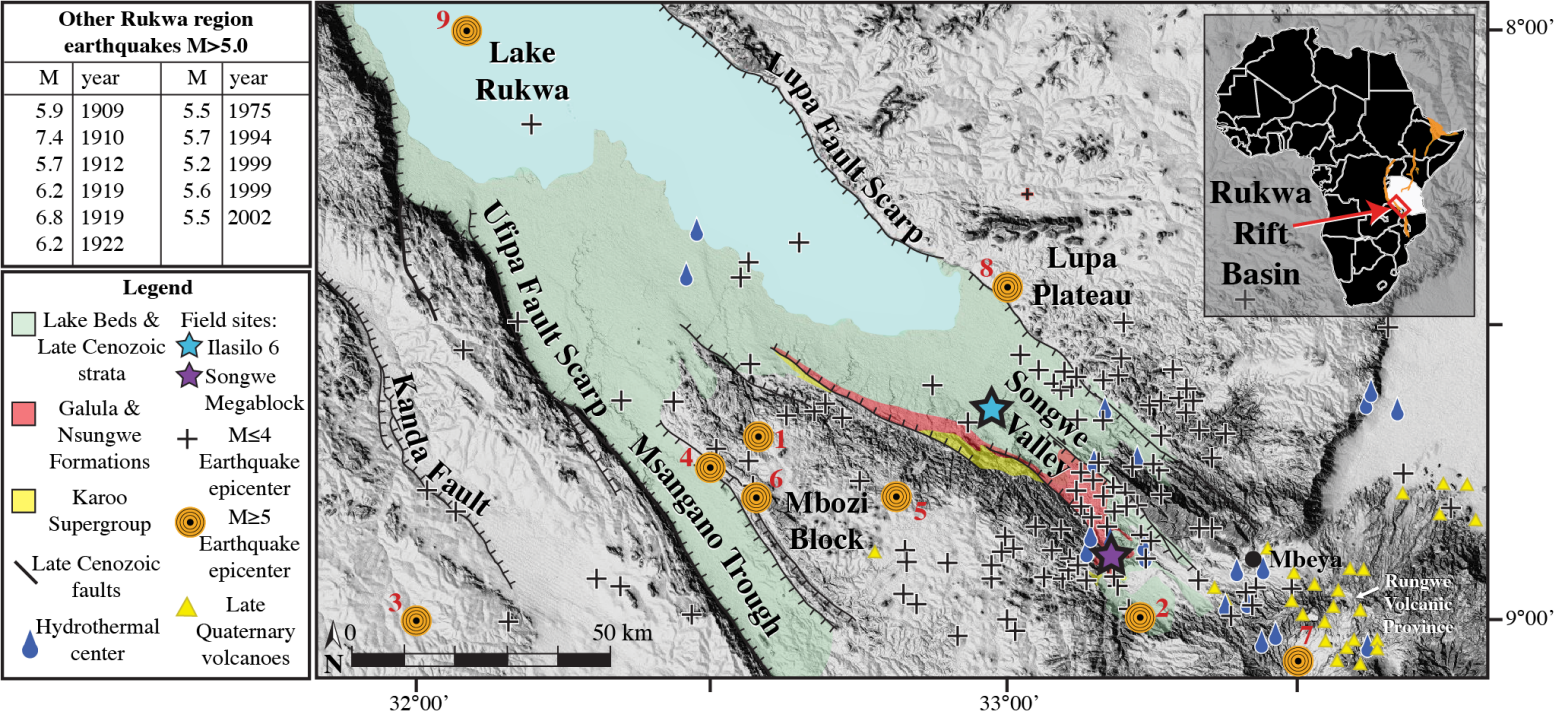
DEM map displaying neotectonic elements of the Songwe Valley, Rukwa Rift Basin [11]. Camelbeeck and Iranga’s seismic network recorded 199 microearthquake (M ≤ 4) events in the Rukwa Rift Basin from 1992–1994 [8]. A sample of these epicenters is plotted on the map (**+** symbols) to show the general distribution of activity, highlighting the Songwe Valley as the most seismically active area [8]. Historical earthquake epicenters in the mapped region with M ≥ 5 include: 1–1968, M 5.0; 2–1972, M 5.0; 3–1979, M 5.3; 4–1984, M 5.4; 5–1985, M 5.1; 6–1988, M 5.0; 7–1994, M 5.0; 8–2012, M 5.0; 9–2013, M 5.0. Other comparable earthquakes that plot just off the map are listed in the table [1, 12, 13].

The Rukwa Rift Basin is a nexus of tectonic activity (Fig 1), and with one of the thickest continental sedimentary successions in Africa, it records repeated rifting, volcanism, and sedimentation from the Permian to Recent [6, 7]. At its southern extremity the Rukwa Rift Basin splits into the Msangano and Songwe (study area) valleys (Fig 1). Over the last century, the Songwe Valley has been characterized by particularly high micro-seismicity, moderate and strong earthquakes (5 ≤ M ≤ 7.4), and significant Holocene fault movements [6, 8–10]. Related to Miocene-Recent rifting, the Lake Beds Succession (LBS) is the youngest deposit in the Rukwa Rift Basin, comprised of semi-consolidated volcaniclastic siltstones, mudstones, sandstones and conglomerates deposited by fluvial deltaic, alluvial, and lacustrine processes.

Numerous soft-sediment deformation features of possible seismic origin have been identified at a variety of different localities spanning the Paleozoic-Recent sedimentary succession in the Rukwa Rift Basin. Indeed, a number of other workers have noted the presence of such secondary sedimentary features, particularly in the Cretaceous-Paleogene Red Sandstone Group strata, and they have also interpreted a seismic origin for many of these features [14–16]. This paper is the first to report soft-sediment deformation features from the Pleistocene-Recent Lake Beds Succession in the rift. We have documented numerous such features, of centimeter- to dekameter-scale and varying character, in the Lake Beds Succession at many stratigraphic levels and at numerous localities in the southern portion of the Rukwa Rift where we have concentrated our research. Here we focus on two particularly spectacular occurrences of soft-sediment deformation exposed at two, stratigraphically correlative outcrop localities ~35 km apart (Ilasilo 6: 502917 E 9044472 N; and the Songwe Megablock Site: 522904 E 9015130 N; zone 36, ARC 1960 datum; Fig 1).

## Methods

### Permits

The Tanzanian Commission for Science and Technology and the Tanzanian Antiquities Unit granted us permission to carry out our field studies and to take samples. Our field studies did not involve endangered or protected species.

### Fieldwork and Analyses

Fieldwork was conducted in the southern Rukwa Rift Basin, Tanzania (Fig 1), during the Austral winter, from 2012–2014. Outcrops were evaluated using standard sedimentologic techniques. For example, stratigraphic sections were measured with the aid of a Jacob’s staff and Brunton compass.

Data analysis was carried out at James Cook University, Australia. Sediment size and dispersion was measured on a Mastersizer 2000 via laser diffraction, capable of measuring particles of 0.02–2000 μm diameter. Five sediment samples were analyzed and corresponding liquefaction potential was modeled from the recorded data and used to create gradation curves. Sediment samples containing fossilized organic material from 1.5 m below the deformed horizon at Ilasilo 6 and in situ fossilized reed fragments from the megablock siltstone unit of the megablock complex at the Songwe Megablock Site were dated using the Accelerator Mass Spectrometry (AMS) method by Beta Analytic Inc. The samples were pretreated with an acid wash, and conventional radiocarbon ages were corrected for total fractionation effects and rounded to the nearest 10 years per conventions of the 1977 International Radiocarbon Conference. Calibrated ages were calculated using the IntCal13 database [17].

## Large-Scale Soft-Sediment Deformation Features

### Description

#### Megablock Complex

**Fig 2:**
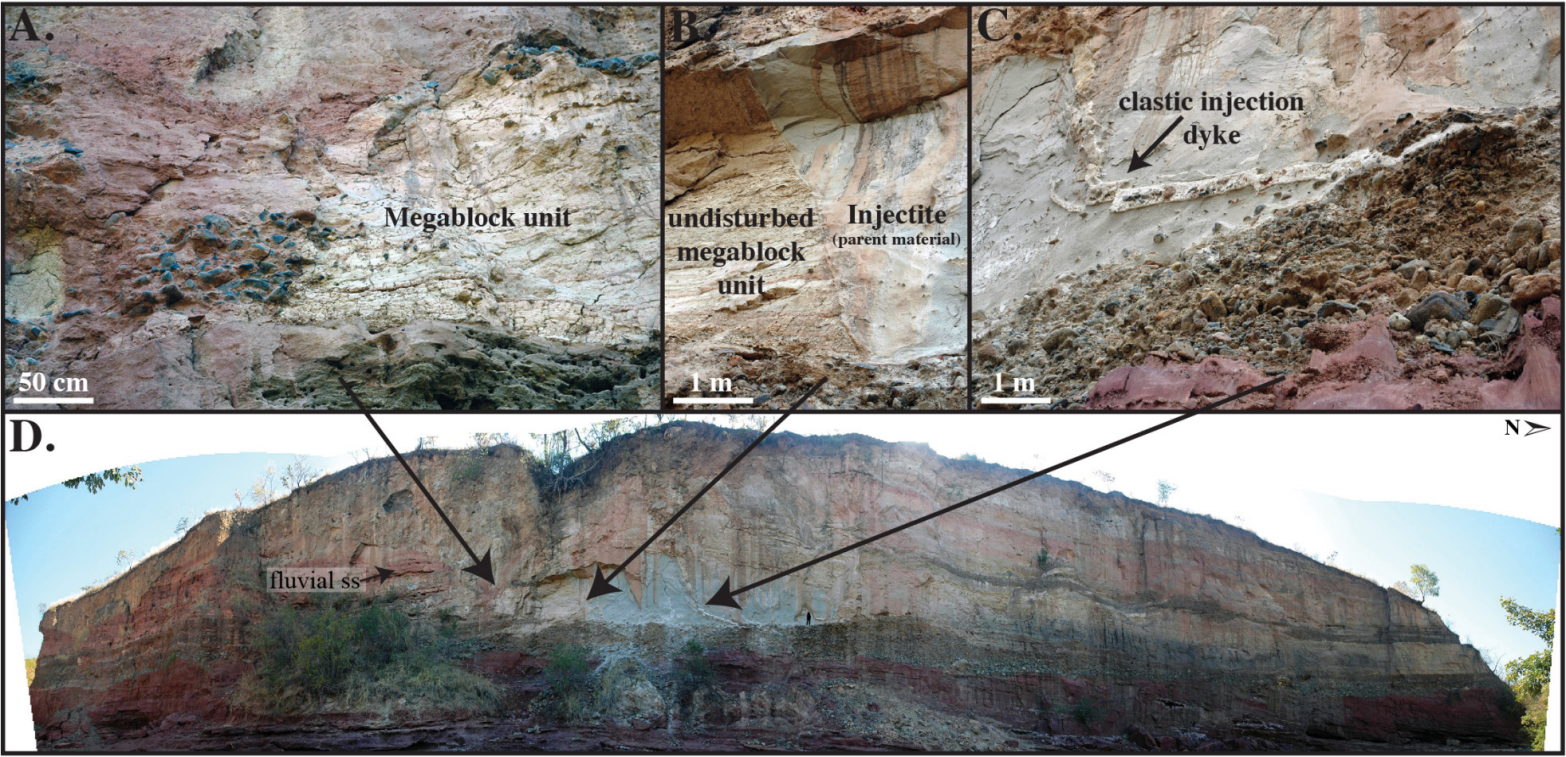
Field photographs of the megablock complex at the Songwe Megablock Site. (A) Megablock unit and offset conglomerate unit. (B) “Blowout” fault bounding the left side of the injectite. (C) Clastic injection dyke emerging from basal LBS conglomerate. Note the pebbles and cobbles entrained in the dyke, as well as the offset dyke segments. (D) Panoramic photograph of injectite complex outcrop, highlighting its position within undeformed, horizontal Lake Beds strata. Person is for scale on the lower right of the injectite.

**Fig 3:**
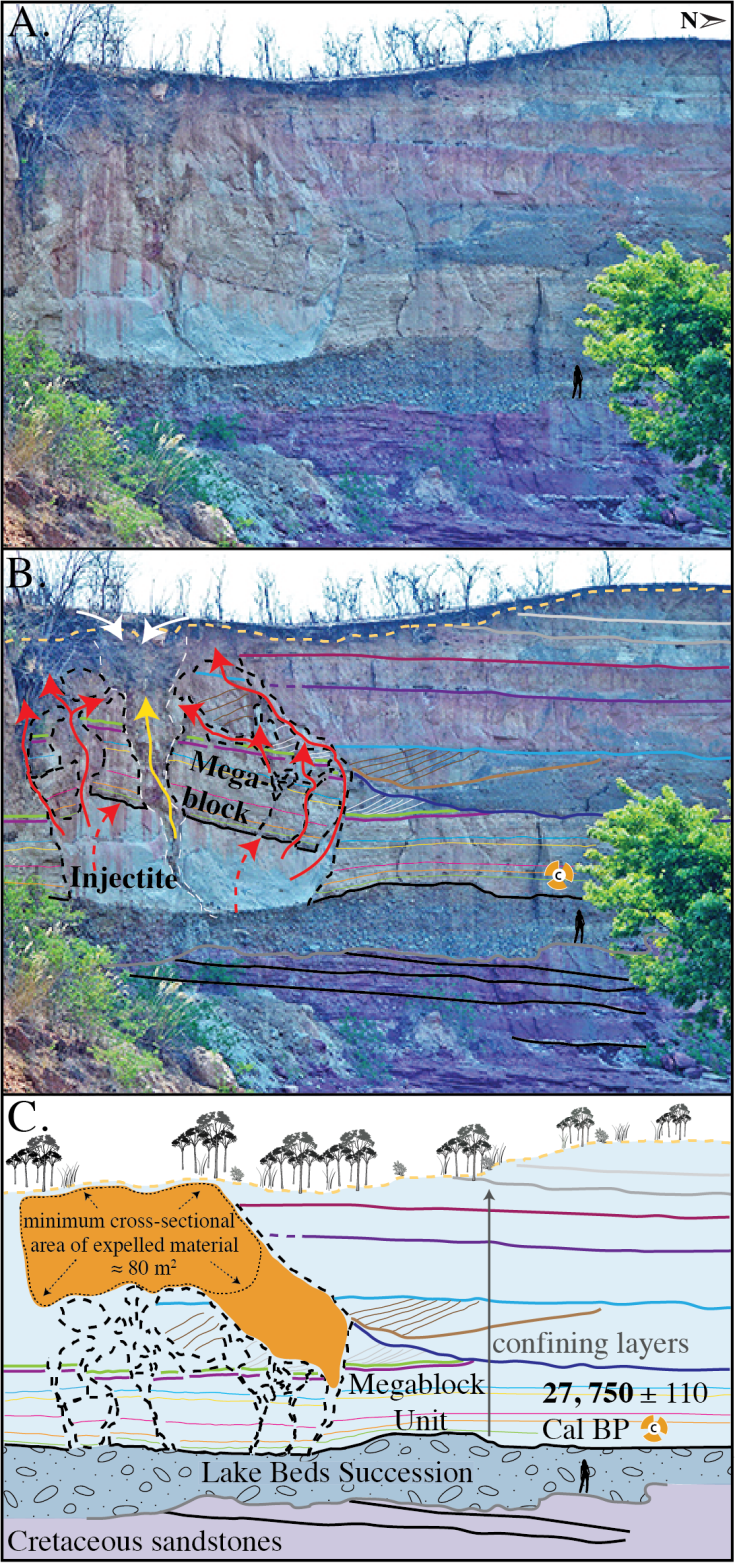
Outcrop exposure of injectite and megablock complex at the Songwe Megablock Site. (A) Photograph of megablock complex outcrop. (B) Trace of intact stratigraphy and displaced megablock. Red arrows indicate flow of injectite material. Yellow arrow indicates path of clastic injection dyke. White arrows represent surface alluvium and clasts of the sidewall infilling the top of the clastic injection dyke. (C) Reconstructed megablock in original stratigraphic position. An estimate is made of the cross-sectional surface area of material ejected onto the surface after formation of injectite and vertical displacement of megablock.

Here we describe for the first time a 50 m-tall cliff face exposure of a spectacular, large-scale (10 m-tall x 20 m-wide in cross-sectional surface area), soft-sediment deformation feature that we term the ‘Songwe Megablock Complex’ (Figs 2 and 3). The Songwe Megablock Site is characterized by an angular unconformity that separates the Upper Pleistocene, upper Lake Beds Succession from underlying, undeformed Cretaceous sandstones that gently dip consistently across the outcrop (~311°, 14° NE). The base of the Lake Beds Succession consists of an upward fining, polymictic, pebble-cobble orthoconglomerate. This unit preserves weak horizontal bedding, remnant trough cross-bedding, weak pebble imbrication, and no obvious deformation. Above the conglomerate a buttress unconformity separates red, dominantly coarse-grained Lake Beds strata to the south (left of the megablock complex in Fig 2D) from dominantly fine-grained Lake Beds strata to the north (right of the megablock complex in Figs 2D and 3), which hosts the megablock complex. The coarse-grained strata is comprised of a basal conglomeratic unit, and overlain by repeated, interbedded sequences of fine-medium sandstone at the base with 1–3 mm pumice clasts, fining upwards into ash-rich siltstone with floating clasts, including ≤ 5 cm metamorphic and caliche pebbles. To the north, the fine-grained units are characterized by finely laminated, tuffaceous siltstone (the strata from which the megablock was derived), and were deposited within an erosionally incised depression that formed along the buttress unconformity surface, against which the fine-grained facies thin. Continuous horizons of thin (~10–20 cm) white cross-bedded ashes are present near the top of the outcrop, along with several massive, color banded, ash-rich siltstone horizons above this (Figs 2 and 3).

A large-scale, soft-sediment deformation feature dominates the finer-grained Lake Beds Succession facies. Deformation is contained between the buttress unconformity and the top of the modern-day cliff face. A 10 m-tall x 20 m-wide block of intact siltstone from the unit immediately above the basal conglomerate appears to have been uplifted ~10 m. The block is supported by a massive, fine-grained foreign body of tuffaceous material, not correlative with the laminated strata on either side of the soft-sediment deformation. This same ‘parent material’ that supports the megablock extends upwards via irregular dykes that surround the displaced blocks of bedded, cohesive sediment. A 5 cm-wide clastic injection dyke emerges from the top of the basal Lake Beds Succession conglomerate, crosscutting the intrusive sediment and uplifted megablock, where it expands gradually into a “V”-shape, up to 2.5 m in diameter at the top of the outcrop. The lower dyke contains metamorphic cobbles (4–20 cm diameter) from the basal Lake Beds Succession conglomerate and abundant pumice clasts (Fig 2C). Metamorphic cobbles and 5–25 cm clasts of the megablock unit infill the uppermost “V”-shaped dyke.

#### Asymmetric Recumbent Folds

**Fig 4:**
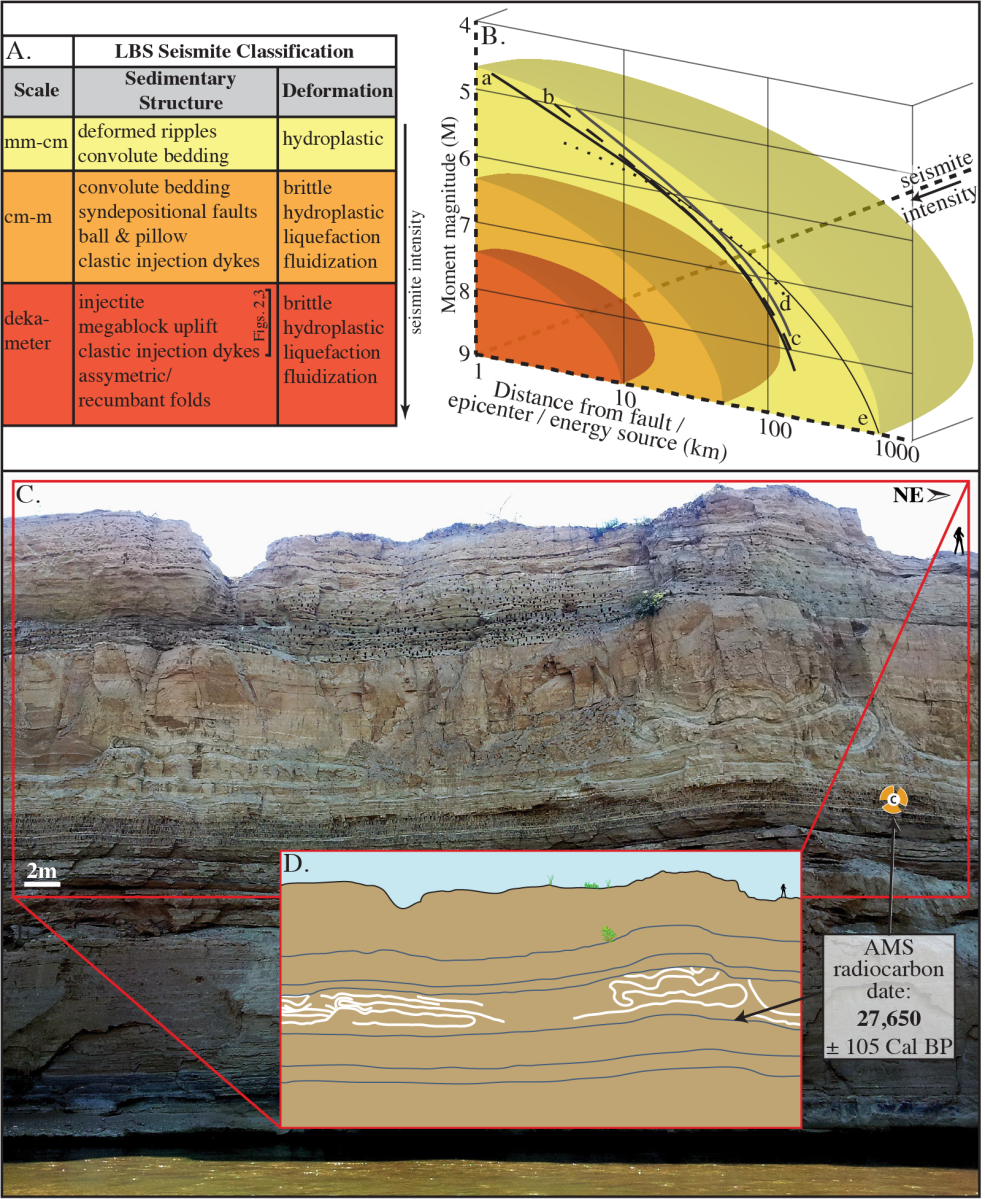
Classification and genetic relationship of liquefaction features in the Songwe Valley. (A) Classification scheme for seismites in the study area (after [18, 19]). (B) Model relating qualitative seismite intensity (refer to Fig 4A) to distribution (distance from fault/epicenter/energy source) and earthquake magnitude. Curve a: upper bound from energy source for worldwide, shallow focus earthquakes [20]; curves b-c: bound from fault [21]; curve d: bound from fault for earthquakes 5.5 ≤ M_s_ ≥ 7.1 [21]; and curve e: bound from epicenter for worldwide, shallow focus earthquakes [20]. The seismite intensity scale (Fig 4A) reported here describes the soft-sediment deformation features recorded from the Lake Beds Succession only; however, the curves reported in part B are global averages. (C) Asymmetric/recumbent folds at Ilasilo 6, illustrated in (D).

Thirty-five km to the northwest of the Songwe Megablock Site, at a locality called Ilasilo 6, dekameter-scale asymmetrical, recumbent folds occur in a 3 m-thick, horizontally bedded, tuffaceous siltstone unit (Fig 4C and 4D), stratigraphically correlative to the deformed horizon (the megablock complex) at Songwe. The folds are overlain and underlain by undeformed, horizontally bedded, volcaniclastic siltstone units of the same lithology. The asymmetric, recumbent fold crests are systematically directed to the southwest. The deformed horizon is truncated by the overlying siltstone, cutting off the tops of the folds.

### Interpretation of Large-Scale Soft-Sediment Deformation Features

#### Megablock Complex

**Fig 5:**
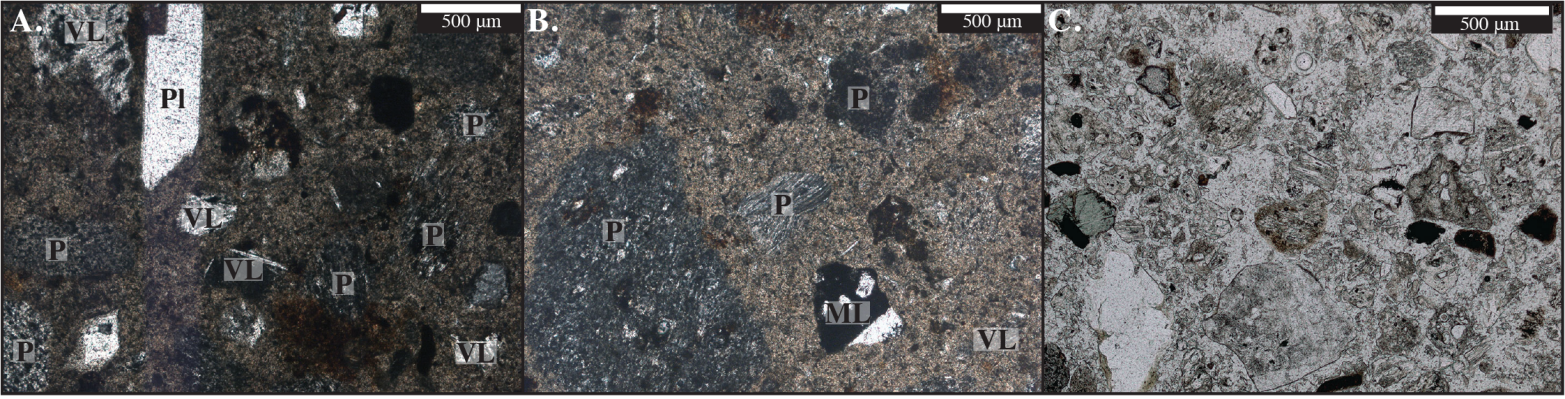
Thin section images from megablock complex samples. (A and B) Thin section of the clastic injection dyke in plane polarized light (PPL), containing abundant pumice and ~90% glassy fragments. (C) Thin section of grain mount of injectite material in PPL. The injectite material is composed primarily of volcanic glass, but also contains few dense mineral grains, including zircon and chlorite. “VL” = volcanic lithic; “ML” = metamorphic lithic; “P” = pumice; “Pl” = plagioclase.

The Lake Beds Succession at the Songwe Megablock Site is characterized by older fluvial-dominated facies and younger lacustrine facies, separated by an erosional unconformity. Prior to the deposition of the lacustrine facies preserved on the north end of the outcrop, a portion of the fluvial units was deeply incised by erosional downcutting. Fluvial incision created a topographical depression, into which lacustrine sedimentation (fine-grained, siltstone dominated facies) records the presence of a network of small, isolated, shallow wetland ponds and lakes. This major decrease in grain size, coupled with a dramatic increase in volcaniclastic sediments (Fig 5) in the lacustrine facies suggests that these units (in which the megablock complex is hosted) were deposited relatively rapidly, preserving reedy plant macrofossils and are associated with a period of intense volcanism and base level rise.

The megablock complex lies on top of the undisturbed, basal Lake Beds Succession conglomerate. This soft-sediment deformation complex displays a unique combination of seismogenically-remobilized sediment coupled with brittle deformation features. It is comprised of (1) an injected body of fluidized volcanic ash that hydraulically displaced (2) an equally sized, semi-consolidated block of intact strata (megablock); both of which were subsequently intruded by (3) a clastic injection dyke (Figs 2 and 3). The buttress unconformity between the basal conglomerate and sealing siltstone above (unit from which megablock was derived) likely served as an initial conduit for lateral flow of fluidized sediment. The parent unit is not found intact or in its original stratigraphic position anywhere along the exposed cliff face. The injected material is lithologically similar to the megablock unit, so we invoke horizontal flow from a laterally correlative unit as a source for the injected parent ash/water slurry (sensu [4, 22]). The ash-dominated parent material was remobilized when pore fluid pressure rose above the hydrostatic pressure gradient [11]. When pore fluid pressure also rose above the fracture pressure gradient of the sealing siltstone unit, the confining layer was hydrofractured into an enormous megablock, along with smaller blocks, of intact strata, bound by reverse “blowout” faults [23] (Fig 2B). Once fluidized, the parent material appears to have been remobilized and driven laterally to the site of the megablock complex by overpressure, where it then hydrofractured, intruded, and hydraulically lifted the overlying volcaniclastic siltstone, similar to the popping of a champagne cork. The megablock and smaller blocks of consolidated, undeformed siltstone were transported upward as the fluidized parent material from below was forced upwards into zones of weakness, flowing around the cohesive megablock(s) towards the paleosurface (Fig 3B).

We interpret strong ground shaking due to seismicity as the source of pore fluid overpressuring, leading to liquefaction and fluidization of the tuffaceous sediment, as discussed in detail below. Cyclic stresses (i.e. seismicity) and/or aftershocks [24] allowed for the overprinting of a brittle structure (clastic injection dyke) on ductile structures (fluidized parent unit and associated megablock), as the sediment was re-deformed after regaining strength following initial fluidization. The clastic injection dyke was formed after the uplift of the megablock complex, as evidenced by its superposition on both the injectite and megablock. Brittle fracturing of the dyke demonstrate that its emplacement was soon after the megablock uplift event, before the fluidized parent material fully dewatered, when post-seismic settling ultimately compacted the injection dyke and led to its deformation. We interpret the upper “V-shaped” portion of the clastic injection dyke as a blowout cone (sensu [25, 26]), representing the top of the dyke where it intersected with the paleosurface. Metamorphic cobbles and 5–25 cm clasts of the megablock unit infill the blowout cone, presumably after injected material was extruded onto the paleosurface [27], and material fell into the cone opening from above.

This megablock complex is significant because it is one of the few continental, outcrop examples of very large-scale soft-sediment deformation associated with injectite features [22, 27, 28], in comparison to the significant subsurface submarine record of sandstone injectite complexes. The megablock complex also stands out because it occurs in rather homogenous, tuffaceous sandstone, compared to the more common scenario of sandstone injectites in mudstone hosts [29–31]. Similarly, the hydraulic *lifting* of the megablock by fluidized injected volcanic ash, defies traditional concepts of downward displaced blocks of coherent sediment, usually due to collapse from overloading, liquefaction, and density inversions. This unique seismite formation illustrates the powerful effects of overpressured systems and active seismicity in this region on near-surface sediments. Consequently, this deformation indicates significant surface stability hazards associated with seismicity in the region, and other parts of the East African Rift System.

#### Asymmetric Recumbent Folds

The large-scale asymmetric, recumbent folds at Ilasilo 6 record hydroplastic soft-sediment deformation in a quiet, shallow lake or pond. The orientation of the folds suggests a very low slope (0.5°-2°) at the time of deformation. The folded horizon is truncated, indicating that the deformed unit was at the sediment/water interface at the time of deformation.

## Discussion

### Identification of a seismic triggering mechanism

Seismites are horizons of secondary sedimentary structures generated close to the surface by earthquakes of great enough magnitude (M ≥ 5 ± 0.5) [20, 32–36] to sufficiently increase intergranular pore pressure and cause liquefaction and/or fluidization of the affected sediment [18]. There are no unequivocal criteria for identifying seismogenic soft-sediment deformation structures, as morphological expression varies widely and is dependent on lithology and depositional history, no two of which are ever the same. However, many authors refer to sets of criteria proposed by Sims [37], Obermeier [38, 39], and summarized by many others, to link deformation structures with seismic events. These criteria generally include: 1) association with seismically active faults; 2) liquefiable sediments; 3) similarity to structures formed experimentally or by recent earthquakes; 4) horizons of deformation that are correlative over large areas; 5) deformed zones surrounded by undeformed strata; 6) deformation that increases in intensity towards the inferred epicenter; and 7) the exclusion of other triggering mechanisms [37, 39, 40].

However, the above conditions are not diagnostic, and often these criteria can also fulfill the requirements for triggers other than seismicity. In contrast, some workers advocate a context-based approach [41–43] to identify a trigger mechanism as either autogenic or allogenic, based on sedimentological and paleoenvironmental setting. In light of the problematic methodology for identifying a trigger mechanism for soft-sediment deformation, we use a combination of published criteria and depositional environment- and deformation style-based evidence to infer a seismic trigger for the large-scale megablock complex and asymmetric, recumbent folds at Ilasilo 6.

**Fig 6:**
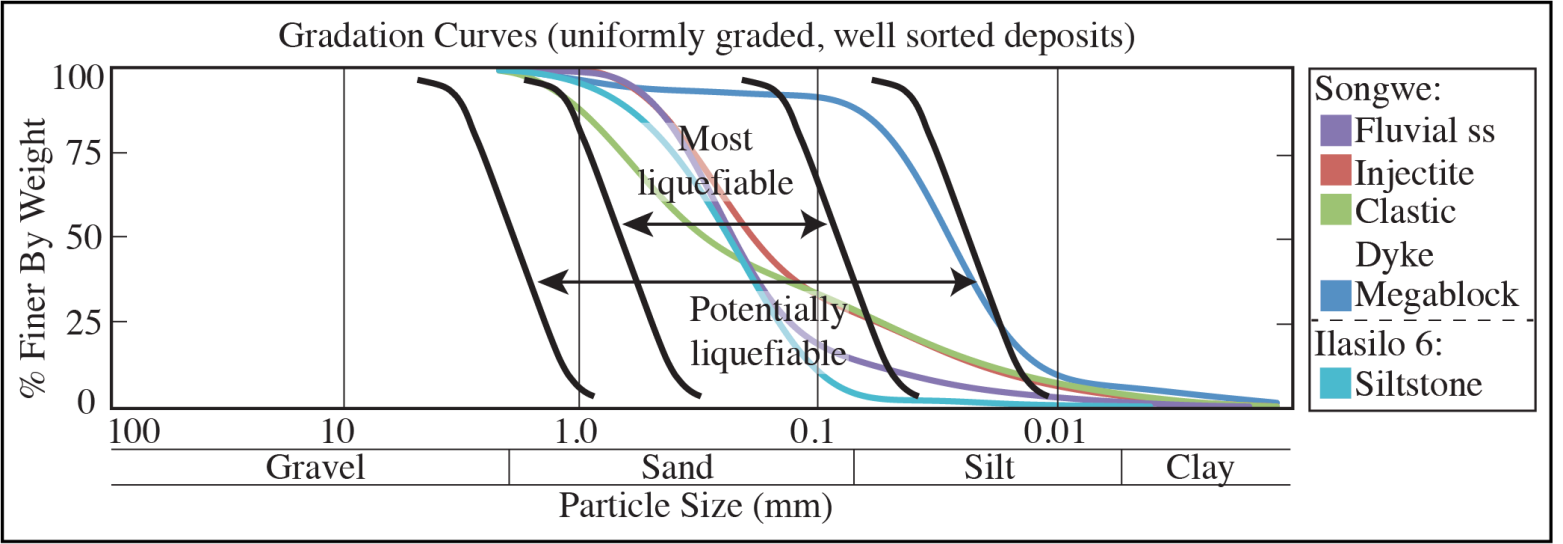
Gradation curves modeling the susceptibility of sediment from the Rukwa Rift Basin to liquefaction [44]. The sealing unit of the megablock complex is represented by the sample labeled “Megablock”. Note that this lithology is the least liquefiable, in comparison to the fluidized parent unit (“injectite”) and clastic dyke material, which plot within the “most liquefiable” zone. Sediment size and dispersion was measured on the Mastersizer 2000 via laser diffraction. The “fluvial ss” sample is located on Fig 2D. This unit represents the fluvial facies incised into by the buttress unconformity and against which the lacustrine siltstones of the megablock complex were deposited.

**Fig 7:**
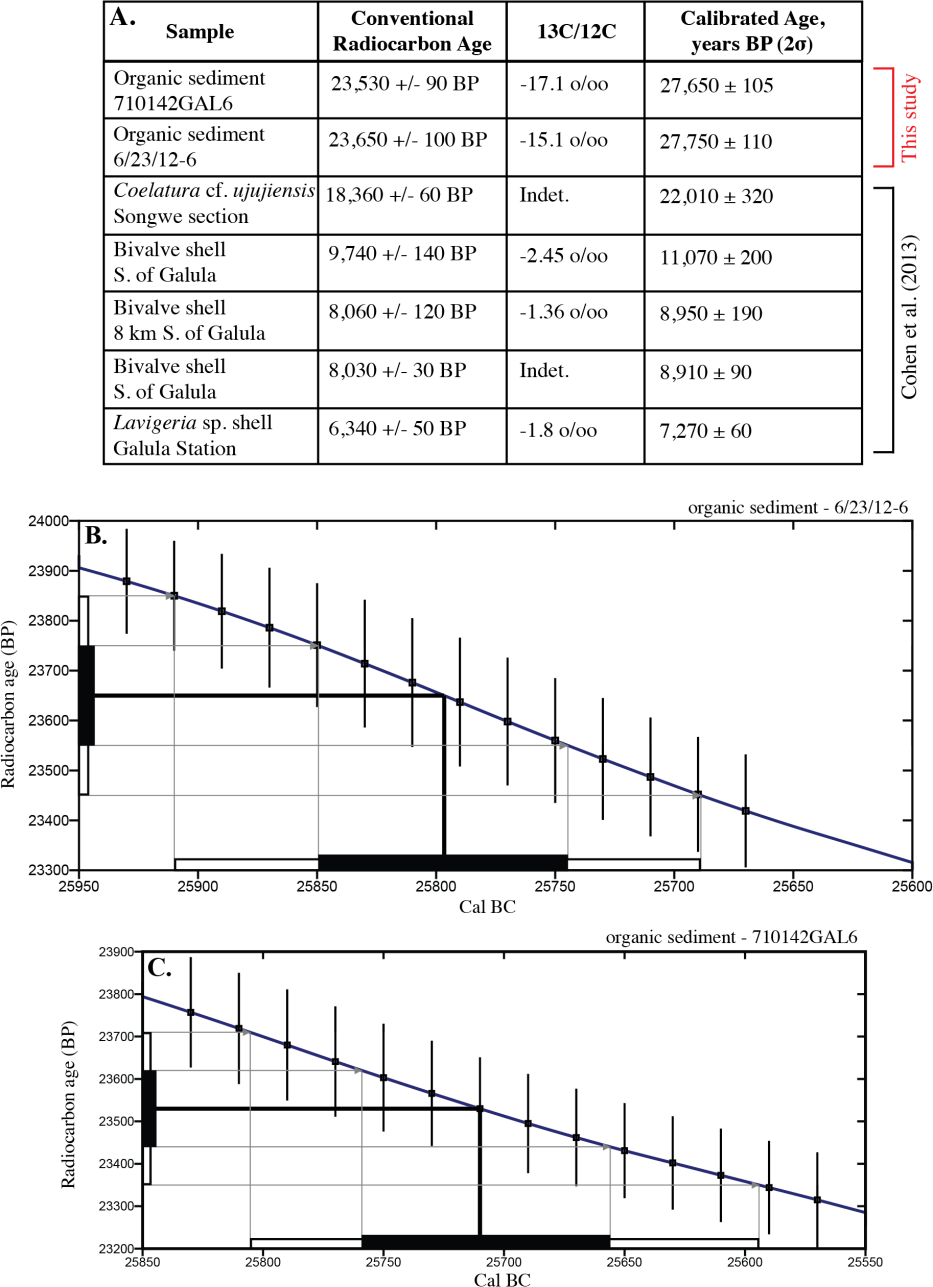
AMS radiocarbon dates from the Songwe Valley region. (A) Dates from the Ilasilo 6 seismite, Songwe Megablock site, and samples from Cohen et al. [45] sourced near Ilasilo 6. (B and C) The intersection of the megablock unit sample (see Fig 3) and Ilasilo 6 seismite sample radiocarbon dates (see Fig 4), respectively, with the calibration curve [17].

**Fig 8:**
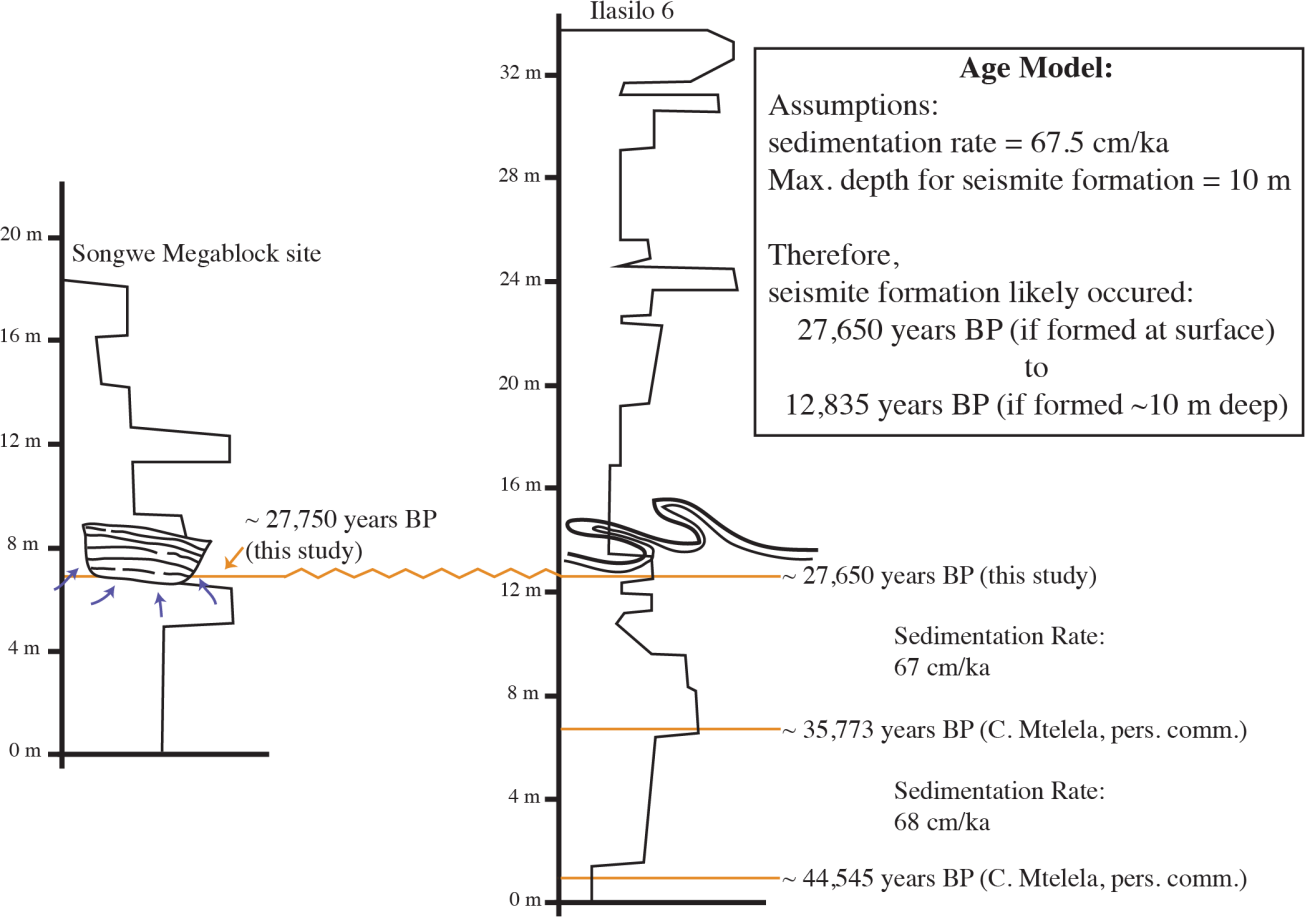
Correlation of Ilasilo 6 and the Songwe Megablock sites, with model approximating age of deformation. The Songwe Megablock Site radiocarbon sample is from an organic-rich layer containing macrofossil reeds of the unit that was fractured and uplifted, forming the megablock (UTM: 522904 E 9015130 N, zone 36, ARC 1960 datum). The Ilasilo 6 radiocarbon sample is from a black, fossiliferous, organic-rich unit ~1.5 meters below the large-scale seismite (UTM: 502917 E 9044472 N, zone 36, ARC 1960 datum). These two indistinguishable radiocarbon dates provide a tie point for the correlation of the two deformed outcrops over 35 km, and support the hypothesis for synchronous deformation of the megablock complex and asymmetric, recumbent folds.

**Fig 9:**
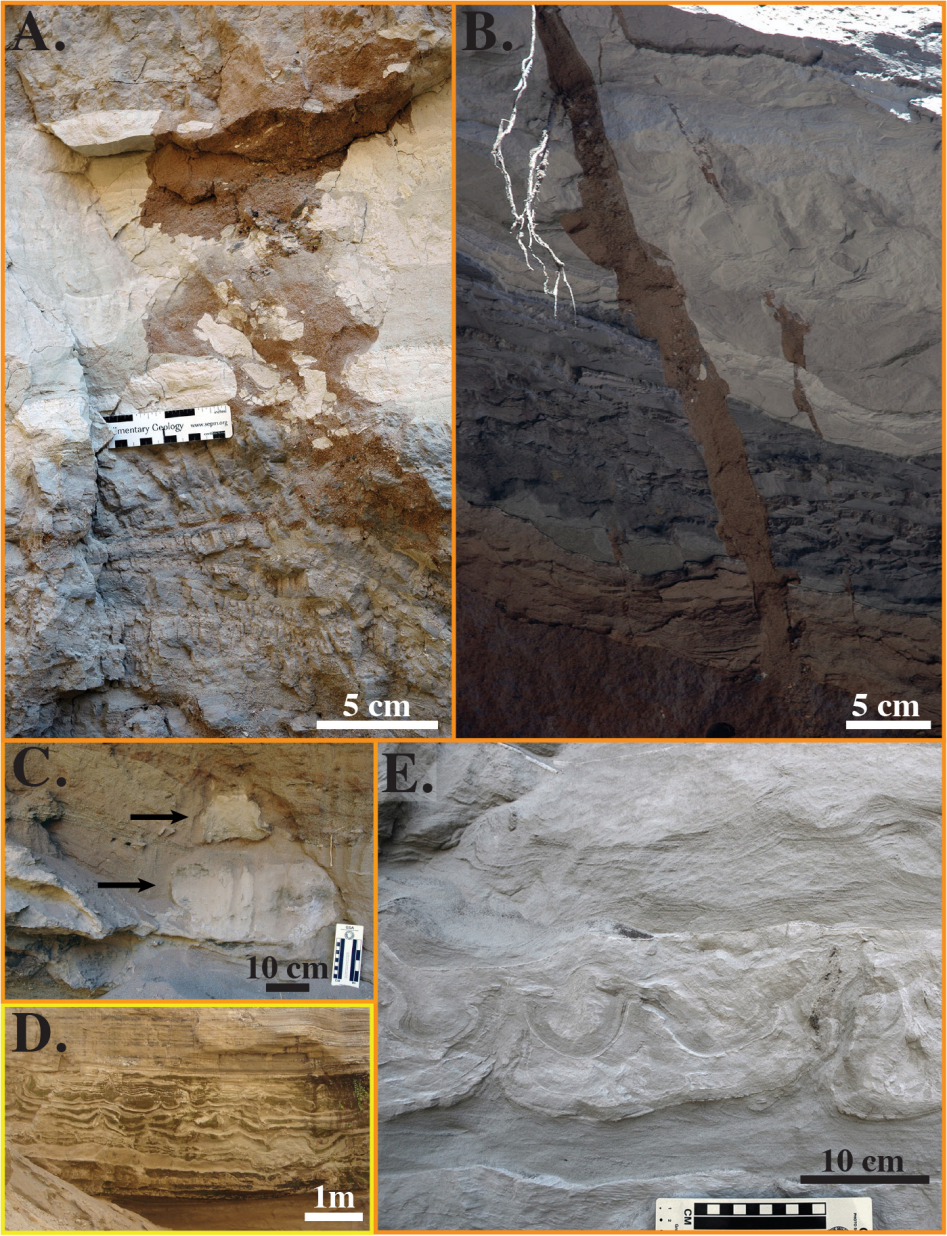
Seismites from the Lake Beds Succession at Ilasilo 6. (A and B) Clastic injection dykes. Vertical movement of clasts of the sidewall rock and sandstone parent bodies present below the field of view of the photographs indicate upward-directed injection. Bottom scale in (A) is in cm. (C) Ball-and-pillow structure. (D) Cm-scale convolute bedding. (E) Convolute bedding, folds, flame structures, and evidence of vertical fluid escape. Refer to Fig 4 for color-coding. Soft sediment deformation features were photographed on vertical, southwest- (A and B), northeast- (C and D), and southeast-facing (E) canyon walls of LBS strata, exposed by down cutting of modern-day rivers.

The following field-based evidence is used to meet the above criteria and to diagnose a seismic trigger for the soft-sediment deformation in the Lake Beds Succession in the southern Rukwa Rift Basin:

1. Deposition of the volcaniclastic, Late Pleistocene-Recent Lake Beds Succession occurred simultaneously with active volcanism and active faulting in the southern Rukwa Rift (Fig 1). Refer to “Implications” section below for details.
2. Grain size analysis suggests the deformed sediment is highly susceptible to liquefaction (Fig 6). The upper surface of the folded horizon at Ilasilo 6 resembles an erosional surface, an indication of liquefaction at the sediment/water interface (Fig 4C and 4D) [43]. Additionally, clastic dykes and the injectite of the megablock complex show evidence of water-escape.
3. Large-scale deformation features are correlated via radiocarbon dates over 35+ km (Figs 7 and 8).
4. Folds at Ilasilo 6 are overlain and underlain by undeformed, horizontally bedded siltstones. The megablock complex is positioned within undisturbed, horizontal bedding and preserves intact, correlative stratigraphy on either side (Figs 2, 3 and 4).
5. Soft-sediment deformation horizons are not only a common and repeated occurrence in the Lake Beds strata, but have also been abundantly recognized in Permian–Paleogene strata of the Rukwa Rift Basin. For example, stratigraphic sections at Ilasilo 6 and elsewhere in the Rukwa Rift Basin record diverse forms of soft-sediment deformation, including: flame structures; cm- to m-scale folded beds; ball-and-pillow structures; syn-sedimentary faults; sand injection features; and m-dkm-scale clastic injection dykes (Fig 9). Clastic injection dykes are most common (n >15), occurring at many localities, where they vary in length from <30 cm to >10 m, and from a few mm to >25 cm in width. Many initiate in sand beds, cut confining horizons of distinct sandstone, mudstone, or siltstone lithologies, and contain cm-scale angular fragments of the side wall rock.

The circumstantial evidence presented above strongly suggests a seismic triggering mechanism for the large-scale soft-sediment deformation in the southern Rukwa Rift Basin. However, because seismite criteria can be ambiguous, we also consider other likely triggering mechanisms. Sediment loading can be ruled out because of the homogeneity of tuffaceous, silt-sized lithology that dominates the outcrops at both localities, eliminating overloading and inverse density gradients as triggers. Additionally, truncation of the folds at the top of the deformed unit at Ilasilo 6 indicates formation near the sediment/water interface [46]. Likewise, the megablock complex is positioned to within ~10 m of the paleosurface. At the Songwe site, the intrusion of massive sediment from an underlying or stratigraphically equivalent parent body demonstrates horizontal and vertical injection as the driving mechanism. At both localities we interpret the facies to represent a flat lying lake floor unit. Vertical displacement of the megablock and block-bounding faults displaying reverse orientation also rule out a trigger originating from the sedimentary depositional environment itself. At Ilasilo 6, the directional element within the folding suggests the aid of a downslope force. Assuming paleo-slope was similar to the present day maximum lake floor slopes (0.5°-2°), and considering fold asymmetry, these seismogenic slump folds were likely gravity-driven, related to slope failure, and seismicity is again the favored mechanism to have reduced sediment shear strength to allow for such hydroplastic deformation [47–49]. Radiocarbon age correlation with the seismogenic Songwe megablock complex supports this interpretation. Storm waves are also discarded as a trigger mechanism because the finely laminated, horizontally bedded siltstone strata reflects relatively still-water conditions, and there is no evidence of tempestites, storm currents, or gravity-driven density currents. It is possible that other processes that produce high fluid pressures were present, and enhanced by seismicity. For example, abundant hydrothermal vents exist throughout the Songwe Valley and may have intensified overpressure, increasing the risk for seismically triggered liquefaction in the Rukwa Rift Basin. In summary, the large-scale dimensions; highly liquefiable, low energy, tuffaceous, lacustrine lithologies (Figs 2, 3 and 6); stratigraphic recurrence; regional extent; deformation morphology; in combination with proximity to active faults; and fulfillment of established seismite criteria, suggests deformation triggered by strong seismic shocks for the features described in this paper.

Large-scale (hundreds of meters to hundreds of kilometers) clastic injectite networks, extrudite deposits, load casts, and slumps have been previously identified, and their generation has been attributed to thermal destabilization [50], fluid overpressure due to rapid sedimentation [51], catastrophic triggering mechanism such as seismicity, subaqueous landslides, and bolide impacts [52, 53], lateral pressure transfer [53], etc., although the triggering mechanisms are not well understood [53]. The megablock complex we describe here is similar to these previously reported deformation structures only in scale, but differs in that it is the first seismically generated injectite complex that features uplifted megablocks of intact sediment, in non-marine strata, which represents a process and variety of seismite not previously recognized. This spectacular, large-scale deformation feature suggests intense, Late Pleistocene to Recent seismic shaking in the Rukwa region.

## Implications: Large magnitude earthquake risk

### Age Estimate for Deformation

The Songwe megablock complex and recumbent folds at Ilasilo 6 likely occur along the same stratigraphic surface, as indicated by two radiocarbon dates (Figs 7 and 8), signifying synchronous deformation. An age model (Fig 8) suggests that deformation and related seismicity occurred in the Late Pleistocene, between ~ 27,650 ± 105 and 12,835 ± ~100 years ago (based on sedimentation rates calculated from radiocarbon ages from a stratigraphic section at Ilasilo 6; Fig 8). The upper truncation of folds at Ilasilo 6 and the surface breach of the clastic dyke at Songwe imply that the deformed horizon was at or near the paleosurface at the time of liquefaction and fluidization, suggesting a significant seismic event closer to ~27,650 years ago. There is documented crustal seismicity at this time, demonstrated by fault scarps, faceted spurs, tilted Quaternary deposits, volcanism, and recorded seismicity [9], as well as by activity of Late Quaternary normal faults in the center of the basin and at the southwestern rift flank [6, 54].

### Earthquake Magnitude Estimate

**Fig 10:**
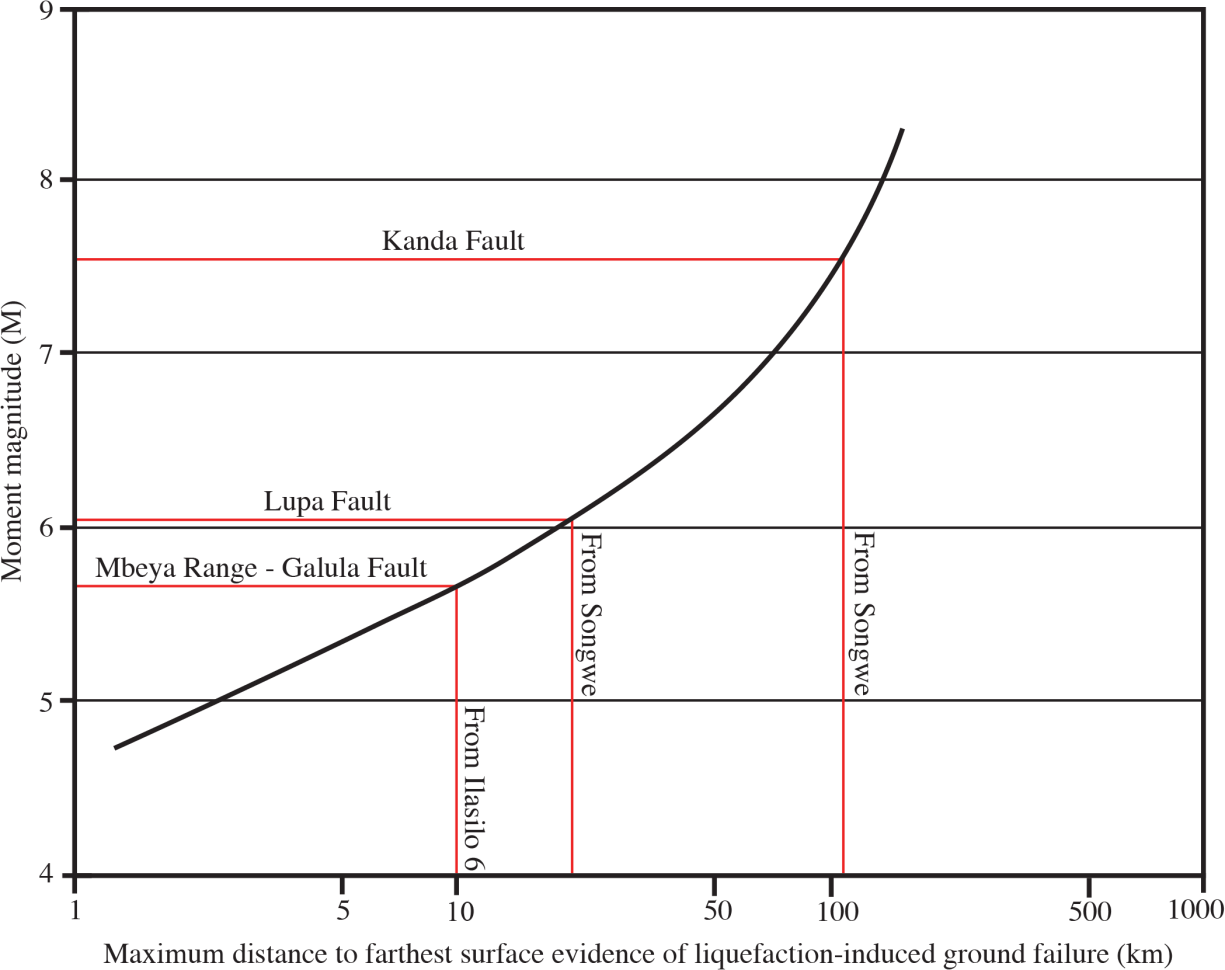
Paleo-earthquake magnitude estimates using the magnitude-bound method. Estimates of minimum paleo-earthquake magnitudes necessary for the formation of liquefaction features at either Ilasilo 6 or the Songwe Megablock Site using the magnitude-bound method of Ambraseys [20]. Distance to farthest evidence of liquefaction was estimated using the distance from either Ilasilo 6 or the Songwe Megablock site to each suspect fault (site furthest away was chosen). Note that this is a minimum estimate of furthest surficial liquefaction features.

We suggest that earthquakes of M ≥ 6 were responsible for the soft-sediment deformation features we report here, based on minimum magnitude capable of liquefaction, and circumstantial evidence, as discussed below. However, relating seismites to earthquake magnitude is equivocal, due to complexities including distance from the causal fault, attenuation of ground motion, and sediment susceptibility to liquefaction, which could not be quantitatively measured here. Many studies [20, 32–36] have reported M 5 ± 0.5 as the lower limit of earthquake energy capable of triggering liquefaction. Empirical relationships have been established between earthquake magnitude and the farthest distance of observed liquefaction [20, 21, 36], and these curves also support this estimate of minimum magnitude. Seismicity responsible for the Lake Beds Succession seismites could have originated at the Kanda Fault (also the proposed source of the 1910 Rukwa earthquake), as nearby LBS deposits record related, Pleistocene–Holocene faulting [55]. One technique for interpreting the strength of paleo-earthquakes, the magnitude-bound method [20], estimates a minimum M ~7.5 earthquake for seismites in the Songwe Valley, if sourced from the Kanda Fault (Fig 10). A second possible seismic source, the active Mbeya Range-Galula Fault, lies just 10–15 km E/NE of the Songwe Megablock Site. This fault system continues beneath Lake Rukwa, where a series of steeply dipping faults are typically syndepositional with the youngest lake sediments. The longest segment of the Mbeya Range-Galula Fault, measuring ~23 km, could be capable of producing an earthquake of M = 6.7–7, as estimated by regression relations of moment magnitude on surface rupture length [56, 57]. A third likely candidate, the Lupa boundary fault, is continuous over 200 km, and has been significantly active, causing major basin forming events (and earthquakes) on intervals of thousands of years [58]. If we assume deformation does not extend pass the Songwe Megablock Site, the magnitude-bound method estimates a minimum earthquake M6 produced by the Lupa Fault for soft-sediment deformation ~20 km away from the source [20] (Fig 10).

Rodríguez-Pascua et al. [4] qualitatively related particular seismite morphologies to earthquake magnitudes. Clastic injection dykes in lacustrine and fluvial deposits were interpreted to form from earthquakes ranging from 5≤ M ≥8 [4]. Cobbles (4–20 cm diameter) from the basal Lake Beds Succession conglomerate contained within the lower meter of the injectite and within the clastic dyke required high injection velocities [4, 22], and this displacement is additional evidence for larger magnitude earthquakes (possibly up to M8 by estimates of Rodríguez-Pascua et al.) [4]. However, we interpret these relationships with caution, as soft-sediment response to seismicity is not only dependent on magnitude, but is also related to ground acceleration, proximity to the source, and other local factors. Minimally, we estimate that the paleo-earthquake magnitude was M > 5 ± 0.5, the minimum energy for triggering liquefaction. However, taking the size of the uplifted megablock and of the Ilasilo 6 folds, their morphologies, and the transport of large cobbles as proxy evidence, we suggest triggering by a higher-magnitude event at that time, especially when compared to the smaller-scale convoluted laminations, clastic dykes, and other seismites we report from underlying and overlying horizons (Fig 9). We estimate a minimum earthquake of M ~6+, taking the most conservative magnitude estimate calculated using the magnitude bound method (from the Lupa Fault). Since 1900, at least 20 earthquakes of M ≥ 5 have been documented in the Songwe Valley region [1, 12, 13] (Fig 1). The sedimentary evidence for recurring, similarly intense palaeoseismic events presented herein emphasizes the potential for damaging surface deformation in the region, and identifies a significant seismic hazard in southwest Tanzania, and indeed throughout the East African Rift System.

## Conclusions

Outcrop exposure of exceptional, large-scale soft-sediment deformation, including the uplift of a bus-sized megablock by fluidized, injected material; fluidization of large cobbles at Songwe; formation of giant, recumbent folds at Ilasilo 6; and numerous smaller scale clastic dykes and seismites, occur over at least 35 km in the RRB. These unique features suggest potential for dangerous surface deformation related to the seismically active East African Rift System. Our documentation provides evidence for M ~6+ Late Pleistocene earthquakes, similar to the M7.4 earthquake at the same location in 1910, extending the record of large-magnitude earthquakes beyond the last century, and contributing valuable data to better constrain recurrence intervals of high magnitude earthquakes in the Western Branch of the East African Rift. Our discovery is consistent with high-resolution seismic reflection data from Lake Rukwa, which provided evidence for high-frequency changes in boundary fault activity that occurs with a periodicity of thousands to tens-of-thousands of years [58]. Documenting new seismite morphologies, such as the one presented here, is essential for understanding surface dynamics and assessing seismic hazards and risks, particularly in rift settings where seismite records remain poorly studied. The nature of the Late Pleistocene to Recent deposits of the Lake Beds strata in the Rukwa Rift Basin, typified by repeated transitions between coarse-grained alluvial/fluvial strata and fine-grained volcanic ash-rich lacustrine strata, provide perfect conditions for land surface deformation and serious hazards associated with seismic shaking, as exemplified by the unique, highly disruptive nature of the megablock complex. With population centers such as Mbeya, Tukuyu, Sumbawanga, and Mpanda all within tens of kilometers from the Songwe Megablock Site or the 1910 M7.4 earthquake epicenter, documenting and predicting the near-surface sediment behavior under seismic stress is critical information for the understanding and modeling of earthquake hazards in rift settings in East Africa.
